# Possible Enantioseparation of Racemic Ribose on Chiral Surface Formed by Adsorption of Nucleobases

**DOI:** 10.3390/life15081160

**Published:** 2025-07-23

**Authors:** Roman Bielski, Michal Tencer

**Affiliations:** 1 Chemventive, LLC, Chadds Ford, PA 19317, USA; 2 MST Consulting, Ottawa, ON K1G 2C1, Canada

**Keywords:** enantioseparation, nucleobases, chiral surface, enantiopure ribose formation, absolute enantioselective separation

## Abstract

The paper proposes a putative prebiotic scenario leading to homochirality in the RNA world. In this scenario, racemic ribose, the only chiral moiety in RNA, was enantioseparated (in its pyranose form) on a chiral surface formed by the adsorption of (prochiral) nucleobases (NBs) on a mineral or metal. Purine bases (adenine and guanine) are more likely candidates for this process than pyrimidine bases because they have more H-bond donors and acceptors. Another possible candidate surface for the enantioseparation of ribose would be formed by the adsorption of nucleobase pairs, e.g., guanine–cytosine (GC). Interactions of ribose molecules with hydrogen bond donors and acceptors of NBs or NB pairs (located on the surface) enforced the orientation of ribose molecules in two directions perpendicular to each other and parallel to the surface. Consequently, the energy of interactions of enantiomers of the sugar with the surface was not the same. Thus, a solvent moving along the surface caused the enantiomers of ribose to move with different rates, resulting in the enantioseparation of ribose in a chromatography-like process. The same process would also separate ribose from other monosaccharides in the mix. Hydrogen bonding between nucleobases was also pivotal in the formation of large homochiral domains on the surfaces.

## 1. Introduction

The following two issues are among the most fundamental unsolved problems of science:

- The origin of homochirality.
- Why is ribose and not another sugar the chiral component of ribonucleic acid?

The first question was probably first formulated by both van‘t Hoff and Le Bel in 1874 in their independently published papers proposing tetrahedral carbon, thus marking the birth of stereochemistry. Interestingly, both authors invoke circularly polarized light (the only chiral influence known at the time) as a possible factor leading to enantiopure compounds.

Beginning at the end of the 19th century, chemists have attempted to demonstrate experimentally the formation of enantiopure or enantioenriched compounds from racemic and/or non-chiral reactants. However, for about a century, there were no acceptable solutions of the problem usually called the “origin of homochirality”. A number of interesting experimental results offering more or less satisfactory approaches have appeared in the last 30 years. The first was the 1995 discovery of the autocatalytic Soai reaction [1]. A decade later, Viedma described a spectacular case of chiral symmetry breaking during crystallization, now known as Viedma ripening [2,3,4]. Recently, Naaman and coworkers achieved excellent results in enantioseparation by crystallization using magnetic substrates [5,6]. This approach has been shown to produce a highly enantioenriched product of relevance to the abiotic synthesis of nucleosides [7].

The second question was best formulated by Albert Eschenmoser. Towards the end of the previous century, he [8] asked “Why did Nature choose a pentose and not a hexose for its nucleic acids? And, given a pentose, why ribose and not one of the other diastereomeric pentoses? And, finally, given ribose, why ribofuranose and not ribopyranose?” Eschenmoser et al. [8,9,10,11,12]. have set out to experimentally study the chemical etiology of the nucleic acid structure. Perhaps surprisingly, pyranosyl RNA turned out to be not only a much stronger base-pairing system than RNA but also one that is more selective with respect to base-pairing mode. Further studies showed that base pairing in oligonucleotide systems containing pentopyranosyl units as building blocks turned out to be stronger Watson–Crick systems than RNA. Therefore, the “maximization of base-pairing strengths within the domain of pentose-derived oligonucleotide systems was not the critical selection criterion” is a rather surprising conclusion. Eschenmoser states that “in principle, a chemical etiology of nucleic acid structure has to reckon with the possibility that the RNA structure might have originated as a consequence of synthetic contingency, not as a result of synthetic variation and functional selection”. However, Banfalvi proposed an interesting idea that β-*D*-ribofuranose fits best into the structure of physiological forms of nucleic acids [13].

The RNA world hypothesis utilizing the enzyme-like catalytic properties of RNA, which is increasingly more accepted [14,15,16], offers a measure of simplicity to the investigation of life’s origin. We think that it also offers an insight into the origin of its exclusively observed homochirality. In both single-stranded and double-stranded RNA, the only chiral moiety is ribose (or its D enantiomer, to be precise). This casts this sugar in the most pivotal role not only in the stereochemistry of RNA strands (including their screw sense) but also the selection of L-amino acids for protein synthesis. In this connection, ribose seems to be the key to the homochirality of life and, indeed, to life itself.

Currently, it is the predominant opinion that the appearance of homochirality predates the emergence of life [17,18,19]. Accordingly, Joyce et al. [20] demonstrated that homochirality is crucial for effective RNA polymerization.

**Figure 1:**
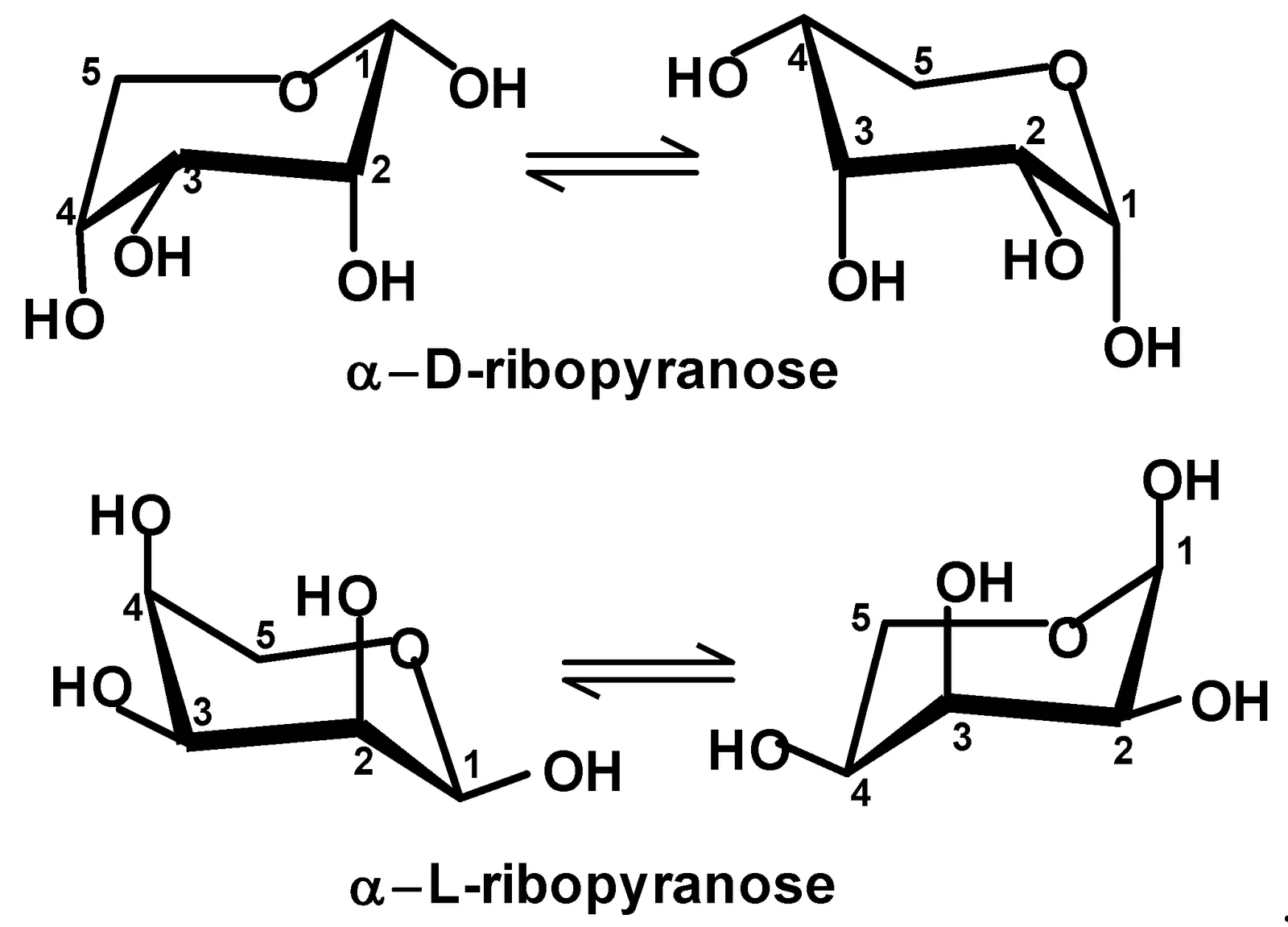
The chair conformers of α-D- and α-L-ribopyranose. Carbon atoms are numbered in accordance with the standard carbohydrate nomenclature, i.e., a carbonyl carbon atom in aldoses carries number 1.

Several years ago, we attempted to look for a perspective that may satisfactorily answer both questions. We proposed a theoretical scenario that could have led to enantiopure compounds starting from a racemate [21,22]. It involves a process we call absolute enantioselective separation (AES). It requires that the molecules of the separated racemate are oriented in two directions perpendicular to each other and parallel to the surface. The orientation can be accomplished as a result of the presence of orienting factors such as an electric field or due to interactions (such as H bonds) with a specific pattern on the surface. A cautious analysis of monosaccharides shows that D,L-ribose is the racemate that is easiest to become separated in the AES process [22]. Figure 1 shows both chair conformations of α-D-ribopyranose and α-L-ribopyranose. If they are oriented as shown (i.e., the anomeric carbon atom {C-1} is on the right-hand side and the ring oxygen atom is further from us), then in one of the enantiomers, all OH groups are directed down from the ring and in the opposite enantiomer, all OH groups are directed up. If these molecules are introduced onto the surface, one enantiomer may form up to four hydrogen bonds with the surface, while the other enantiomer will form no hydrogen bonds with the surface. Such a large difference between enantiomers can be observed only in the case of ribose.

Furthermore, the discussed scenario explains how ribose was separated from accompanying monosaccharides (e.g., other pentoses, hexoses, tetroses, etc.). At the time, we were not able to propose plausible surfaces that may have been available for AES a few billion years ago. In this article, we suggest that adsorbed nucleobases may have been such a surface.

The next step in the proposed scenario requires enantiopure D-ribose from the AES process to be desorbed from the surface, followed by a reaction with nucleobases to form nucleosides and nucleotides.

Initially, attempts to synthesize nucleosides starting from ribose and one of the nucleobases under prebiotic conditions were not successful. Then, the Sutherland group offered a very elegant approach to these compounds, which omits the direct use of ribose and nucleobases [23,24]. Consequently, it seemed that ribose is not a plausible substrate for the abiotic synthesis of nucleosides and nucleotides.

However, over the last dozen years, there have been several reports demonstrating the plausibility of nucleoside formation directly from ribose and nucleobases [25,26,27,28,29,30,31,32,33,34,35,36]. They include processes taking place in aqueous microdroplets, syntheses from phosphorylated ribose and nucleobases, and stepwise nucleotide formation from ribose and nucleobase building blocks. Thus, it seems that ribose is a plausible reactant in the abiotic synthesis of nucleic acid monomers.

**Figure 2:**
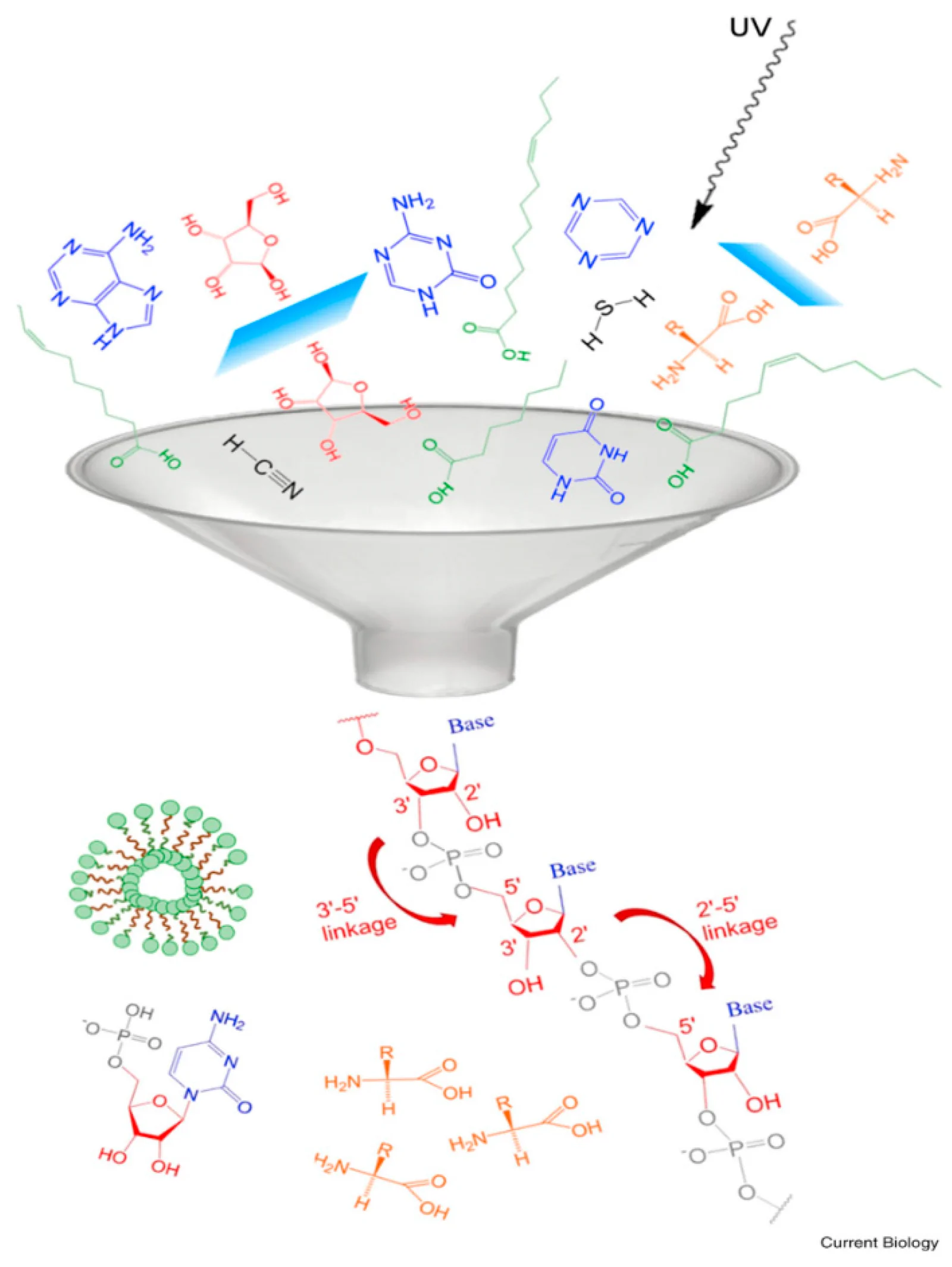
The “funnel” depicting an event leading to the formation of RNA. Reprinted with permission from Pressman et al. [37].

If this had happened a few billion years ago, D-ribose, nucleobases, phosphoric acid, and/or perhaps their derivatives and other simple compounds could have been present in close proximity. Pressman et al. [37] used a funnel as an effective metaphor for such an event (see Figure 2). The funnel may be representative of either spatial or temporal relationships or both.

In consideration of the homochiralty of life, a low probability of its appearance is routinely assumed. Indeed, if all the funnel event participants shown in Figure 2 had independent provenance, the event would have a very low probability. However, if there is a viable process in which one of the participants brings about the formation of another one, the odds of the event significantly improve. Here, we propose that the presence of one or more nucleobases was pivotal in the emergence of one of the ribose enantiomers, although the fact that it was D-ribose rather than L-ribose is still a matter of chance.

## 2. Origin of Ribose and Nucleobases

Before discussing the enantioseparation, let us very briefly look at the current opinions on ribose and nucleobase prebiotic synthesis. Since purines and pyrimidines are relatively simple molecules with no chiral centers, their prebiotic synthesis is rather non-controversial. There is a general agreement that ammonia, formaldehyde, hydrogen cyanide (and its tetramer {1,2-diaminomaleonitrile}), and formamide are likely precursors [38,39].

It is broadly accepted that ribose must have come from some variant of the formose reaction. Discovered in the early second half of the 19th century by Butlerov, it produces a mixture of monosaccharides when aqueous formaldehyde is heated with calcium hydroxide. Today, many variants of the process are known using other bases and other divalent metal catalysts. The formose reaction leads to a very complex mixture of racemic monosaccharides. Particularly interesting are variants developed by the Eschenmoser group [40], Darbre group [41], and Usami and Okamoto [42], since they offer a relatively high concentration of ribose and a small number of additional sugars.

Ribose is not stable in basic aqueous mixtures. Thus, a substantial amount of research was devoted to finding possible methods addressing the problem. In this context, the most important discovery seems to be that boron-containing minerals stabilize ribose [43,44,45,46]. There are also ways nature could have isolated ribose selectively from a carbohydrates’ mixture. For example, Springsteen and Joyce have shown that cyanamide reacts especially rapidly with ribose to form a stable adduct which spontaneously crystallizes in aqueous solution, whereas other monosaccharides do not [47].

## 3. Adsorption of NBs on Surfaces and Induced Chirality

Let us briefly look at the selected studies on nucleobases (adenine and guanine), which underline their properties as being prochiral. As such, nucleobases are compounds that can transform a non-chiral surface into a chiral one when adsorbed on it [48,49,50,51]. “Flat prochiral molecules, which are achiral in the gas phase or solution, can exhibit adsorption-induced chirality when deposited on surfaces” [51]. It must be emphasized that in this context, chirality refers to 2D chirality. Feringa et al. [48] offered an excellent definition of 2D chirality as follows: “A structure is chiral in 2D if it is non-superposable to its mirror image by rotation or by translation in the plane of the surface”.

First, consider the adsorption of NBs on metals. Such studies may explain how homochiral domains could have been formed. Moreover, they may have some direct relevance here since in a prebiotic atmosphere, molecular oxygen (O_2_ and O_3_) was not present, as it is a product of photosynthesis. Thus, free metal surfaces might have been much more ubiquitous then than now, and the metals to consider should not be limited to those which we normally consider “noble”.

The important papers describing research on the adsorption of nucleobases on metals include guanine on gold [52], guanine on silver [53], nucleobases on gold [54], adenine on copper [55,56], and adenine on gold [57]. There are a few observations worth mentioning:

Guanine forms homochiral quartets (tetrads). The R and L (guanine) quartets at room temperature prefer to form homochiral G-quartet networks.

Calculations show that the interaction of nucleobases with a gold surface reaches a maximum when a full monolayer is formed.

Negligible chemisorption was found in calculations of guanine, thymine, and cytosine adsorbed on a gold(111) surface.

Importantly, the homochiral domains of NBs on metal surfaces were shown to be formed.

**Figure 3:**
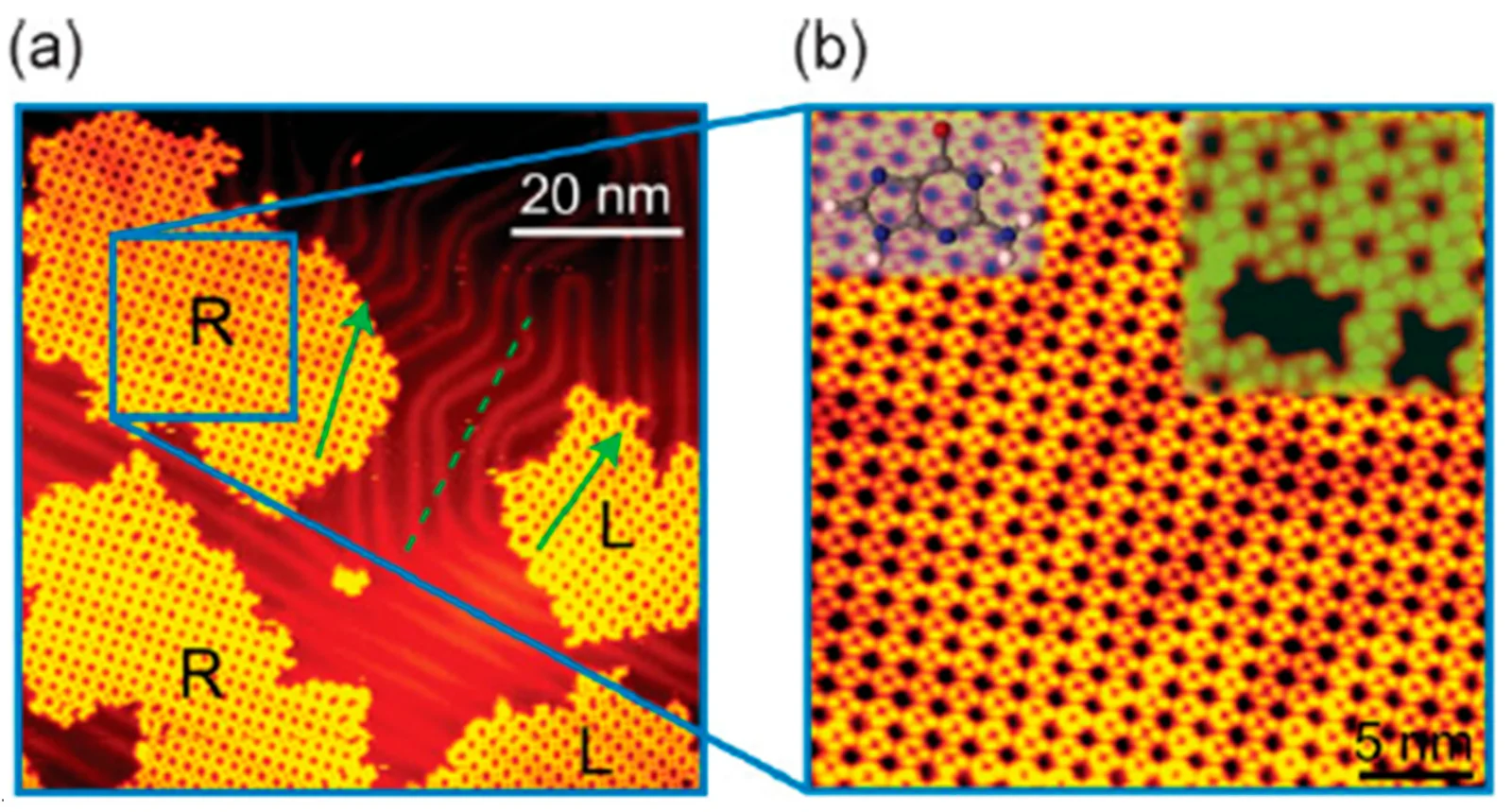
(**a**) An STM image containing mirror phases of enantiomerically pure R and L G-quartet networks. (**b**) A zoom-in of the R G-quartet network; insets: a molecular-resolved STM image of the R G-quartet network (righthand side) and face-up orientation of a G molecule (left-hand side); the. face-down orientation is obtained by flipping the molecule. Reprinted with permission from Xu et al. [52].

Figure 3 shows an example of a relatively large domain of homochiral guanine quartets formed at room temperature. “The stability of self-assembled G nanostructures is predominantly due to intermolecular interactions, that is, hydrogen bonding between individual G (guanine) molecules in this case. The interaction of G molecules with the Au(111) substrate is most likely due to the van der Waals (vdW) interaction, which has a very weak lateral corrugation” [52]. For clarity, it should be emphasized that there is no global symmetry breaking here and larger domains of one chirality will be accompanied with domains of opposite chirality.

The adsorption of nucleobases on surfaces other than metals was also studied. The relevant papers include adsorption studies of guanine on molybdenite [58], guanine on a highly oriented pyrolytic graphite electrode [59], adenine on perovskite [60], adenine on pyrite and silica [61], guanine (and other bases) on hexagonal boron nitride (h-BN) [62], guanine on graphene and black phosphorene (black Pn) [63], adenine and guanine on highly ordered pyrolitic graphite (HOPG) and mica [64], and canonical nucleobases on graphite and MgO [65].

Bhai and Ganguly [66], who studied the adsorption of nucleobases (from air and ethanol) on graphite drew the following conclusions: “The guanine nucleobase showed the superior binding on to the graphene surface as compared to the other nucleobases in the gas phase. The stability of nucleobases to the graphene surface follows as G > A ≥ C ≥ T. The non-covalent interactions (NCI) plot revealed that the nucleobases are physisorbed to the graphene surface”. Very similar are the conclusions of Vovusha and Sanya, who studied the adsorption of nucleobases on MoS_2_, WS_2_, and graphene [67]. Also, Hazhisume [68] reviewed the adsorption of nucleobases (and ribose and phosphate) by some clay minerals. Furthermore, an important review of the self-assemblies of nucleobases at surfaces and interfaces must be mentioned [69]. Note that adsorbed NB molecules have several hydrogen bond centers to interact with other NBs’ molecules (to form homochiral domains) and with ribose molecules.

Most authors do not consider the ability of NB-based chiral surfaces to enable enantioseparation. However, there are exceptions. When studying the self-assembly of adenine on mineral molybdenite, Petersen and coworkers [58] were probably the first to observe that “nucleic acid base molecules can form enantiomorphic surface structures (which may) suggest a mechanism for localized symmetry breaking under plausibly prebiotic conditions.” Sowerby and Heckl [70] also highlight “the possibility that only one stereoisomer of chiral racemate could interact with an enantiomorphic two-dimensional monolayer of base molecules”, which would suggest “the possibility of direct interaction between surface immobilized base molecules and other molecules of prebiotic origin”. They quote Bonner [71], who stated that “chiral surfaces can selectively adsorb chiral stereoisomers of predominantly one configuration.” Also, an important paper by Czech scientists [51], who have shown on-surface asymmetric synthesis, must be noted.

It is worth mentioning the involvement of guanine in the formation of other homochiral networks and chiral separations. Thus, Mali, DeFeyter, and coworkers [72] recently reported on the spectacular emergence and amplification of (homo)chirality in hybrid 2D metallosupramolecular networks formed by a guanine derivative. Furthermore, Sairanova and Gainullina separated racemic chloroalkanes using a new chiral stationary phase based on guanine [73].

## 4. Concept of Ribose Enantioseparation on NB-Coated Surface

**Figure 4:**
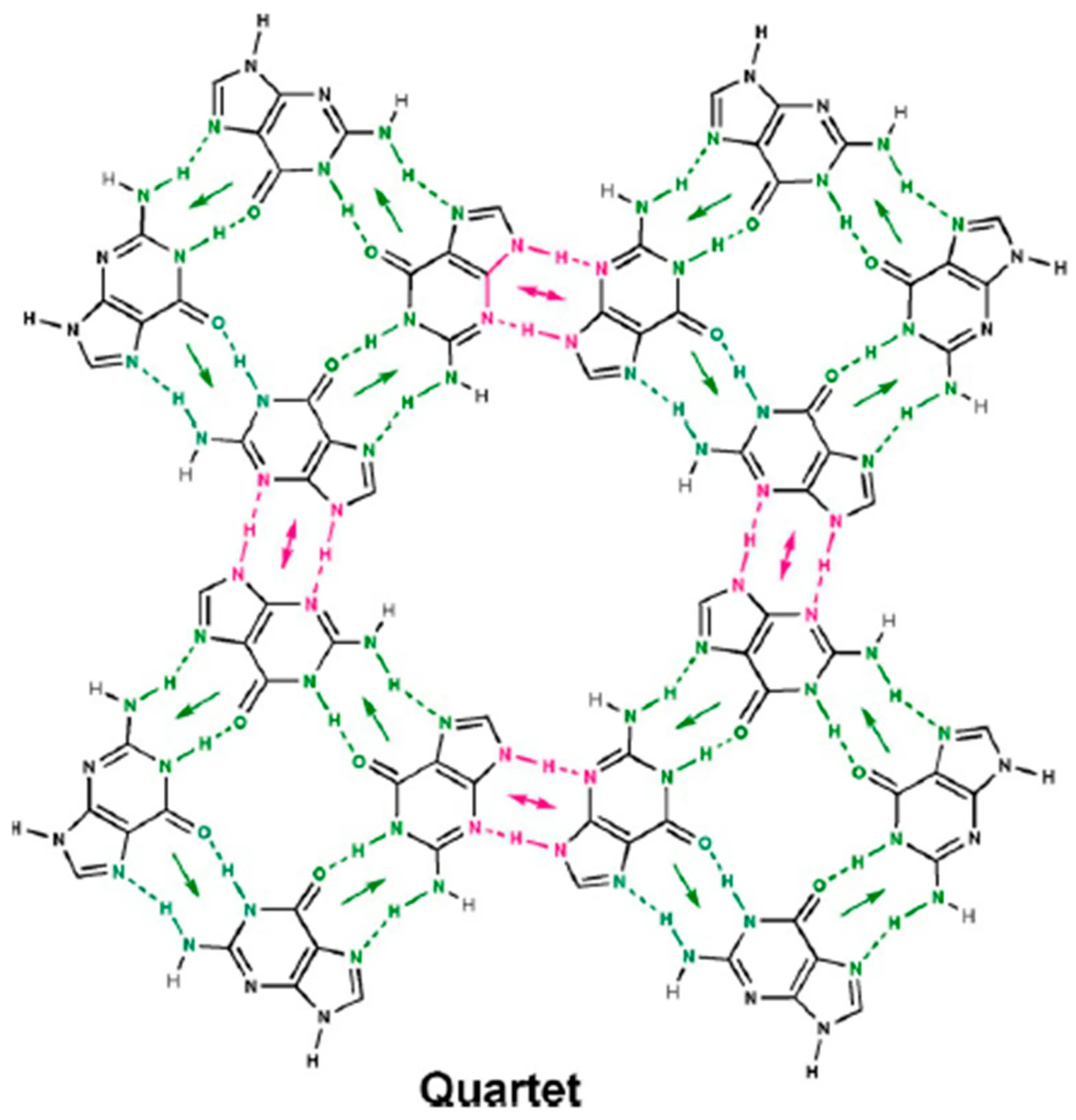
Some of the guanine hydrogen bonds that could have helped form homochiral domains composed of homochiral quartets. Green arrows in the quartet represent the internal hydrogen bonds. Pink arrows in the quartet structure represent the connecting hydrogen bonds. Reprinted with permission from Paragi and Fronseca-Guerra [74].

Both purine and pyrimidine bases are equipped with hydrogen bond donors and acceptors that may help form homochiral domains and also adsorb ribose. Here, purine bases (guanine and adenine) are more likely to be involved than pyrimidine bases due to having more hydrogen bond centers per molecule, e.g., guanine has three HB acceptors and three HB donors, while adenine has three HB acceptors and two HB donors. Thus, one can hypothesize that large homochiral domains were formed on a mineral (or metal) surface by purine molecules (Figure 4). Next, ribose racemate molecules could have been adsorbed on the chiral domains, taking advantage of the hydrogen bonds between ribose molecules and base molecules located on the surface.

The proposed scenario invokes the adsorption of nucleobase molecules on a surface to form a homochiral domain(s) followed by the adsorption of racemic ribose (and its diastereoisomers and other monosaccharides from the formose reaction) on the formed chiral nucleobase surface.

Some time after ribose (and/or other monosaccharides) was adsorbed on the nucleobase homochiral domain (located on a surface), a solvent or solvent mixture started to move along the surface like in planar chromatography. Due to the difference in the adsorption energy between the enantiomers, they moved with different velocities. The same chromatography-like process would also separate other sugars (e.g., racemic pentoses, tetroses, and hexoses) from D- or L-ribose. If the “plate” was sufficiently long, only one of the ribose enantiomers stayed behind and was separated from all other monosaccharides including the ribose opposite enantiomer. Several solvents were considered to have been available abiotically. They included HCN, HCONH_2_, HCOOH, CH_3_COOH, CH_3_CN, supercritical CO_2_, a formate–urea–water mix, “deep eutectic solvents”, etc. [75,76,77,78]. Also, there was a plethora of plausible abiotic surfaces. We do not specify the one nature could have used to adsorb NBs. However, it may be very difficult to determine this, since NBs are rather physisorbed than chemisorbed, and many surfaces could have been appropriate.

## 5. Enantioseparation of Racemic Ribose

In general, enantioseparation can occur when chiral molecules of interest are simultaneously subjected to three orthogonal or near-orthogonal orienting factors [21,79]. Here, one of them is a direction enforced by the interaction of a molecule (ribose, α-ribopyranose, to be more precise) with the surface. Two other directions are parallel to the surface and perpendicular to each other. They are likely to be enforced by hydrogen bond interactions with a pattern on the surface.

Note that the orientation in one of the directions parallel to the surface can be enforced by a strong electric field. Nota bene that the dipole moment of nucleobases is relatively large [80,81]. For example, it is 6.65 D for guanine. We consider the involvement of the electric field rather unlikely, but one should not totally disregard such a possibility.

To understand the interactions ribose–NB, let us examine the structure of ribose. In solution, it exists as an equilibrium composed of α and β furanose (five-membered ring) forms, α and β pyranose (6-membered ring) forms, and a small quantity of the open (aldehyde) form. The situation changes if ribose is chemisorbed on a flat surface using hydrogen bonds. Now, all molecules exist as α-ribopyranose. This is because only α-ribopyranose has as many as four OH groups on the same side of the ring capable of forming four hydrogen bonds with the surface.

Let us draw α-D-ribopyranose (Figure 1). If the ring oxygen atom and C5 are away from us and the C1 carbon atom is on the right-hand side, a surface is below the ribose ring and α-D-ribopyranose has all the OH groups directed towards the surface. α-L-ribopyranose has all OH groups against the surface.

As far as conformations are concerned, there are two chair forms and a more energetic boat form. Out of the two possible chair forms representing this structure, the one with the axial OH substituent at C1 is favored in solution due to the anomeric effect (Figure 1) [82].

In order to orient α-ribopyranose in two directions parallel to the surface and perpendicular to each other, one can utilize two orienting factors. Alternatively, such an orientation can be accomplished when two atoms of ribose interact with two specific points on the surface.

Which structural features of the α-ribopyranose molecule may enforce a certain orientation upon interaction with a patterned surface? Consider the ring atoms. Four of the carbon atoms carry OH groups. The C5 carbon (methylene group) does not. It is thus hydrophobic and may be forced into contact with other hydrophobic points on the surface when hydrophilic interactions involving the molecule come into play. However, such “hydrophobic interactions” would be rather weak. The sixth ring atom (oxygen) differs substantially from all other ring atoms in that it can act as an acceptor of hydrogen bonds.

**Figure 5:**
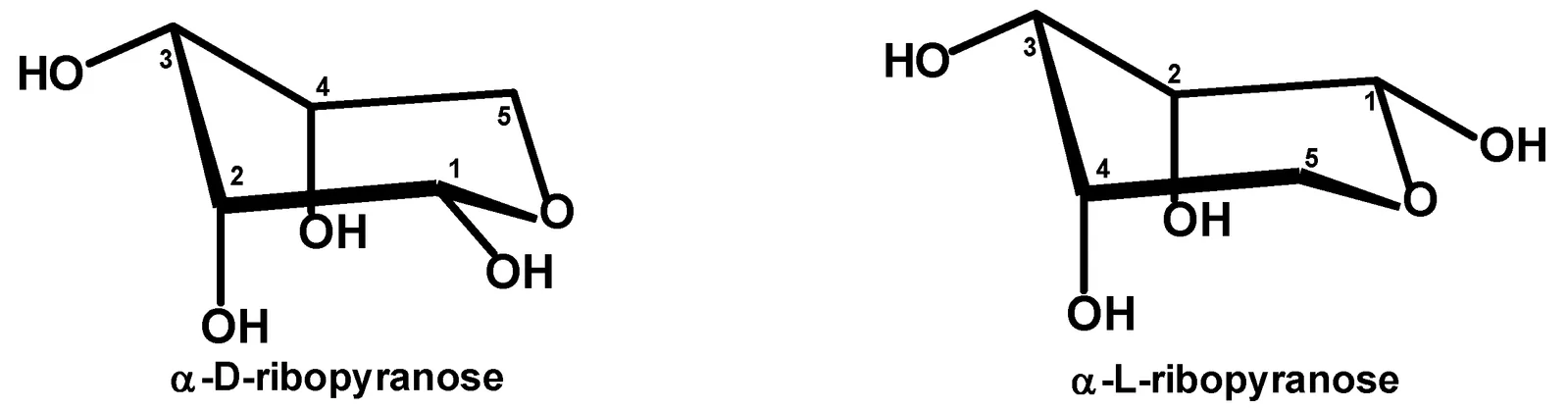
α-D-ribopyranose and α-L-ribopyranose preferred conformations with the ring oxygen atom below all other ring atoms and equatorial OH at C1.

Note that the chair conformers of α-ribopyranose have four ring atoms in a plane, with one atom above and one atom below the plane. To act as a hydrogen bond acceptor, the ring oxygen atom must be below the plane of the molecule (if guanine is below the ribose molecule). Consequently, the hydroxyl group at C1 (anomeric) must be equatorial (Figure 5).

**Figure 6:**
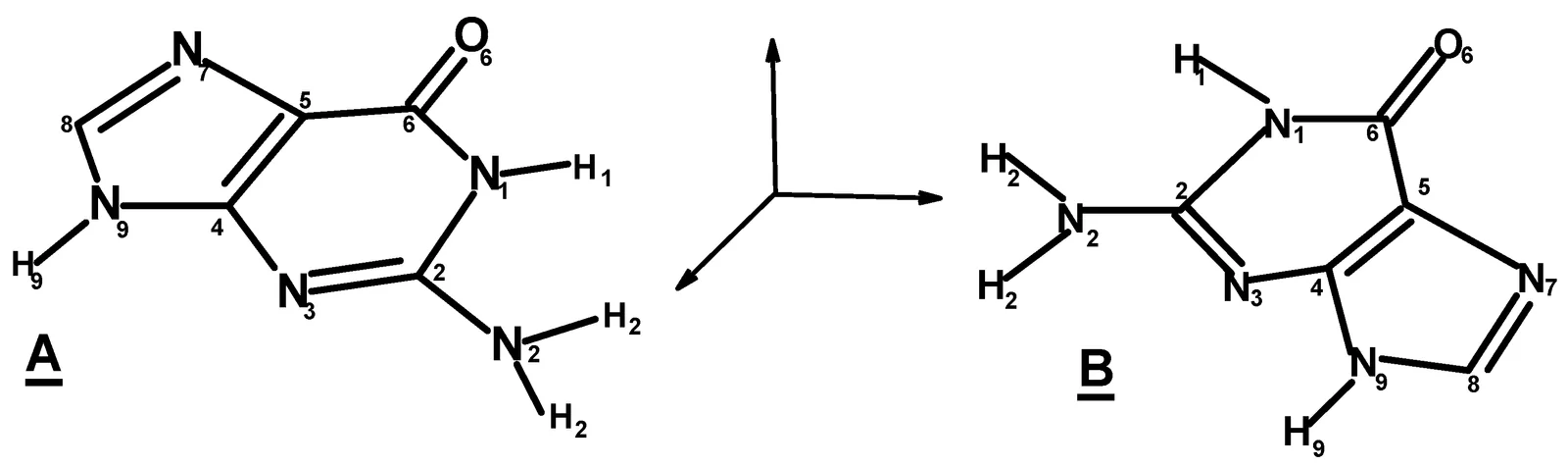
Numbering of guanine atoms. (**A**,**B**) Two possible arrangements of guanine atoms on the surface. This structure is chiral in 2D since it is non-superposable to its mirror image by rotation or translation in the plane of the surface. Note that (**A**) becomes (**B**) as a result of rotation around the C4–C5 bond (not in the plane).

Now, let us analyze a guanine molecule (Figure 6). Guanine is equipped with more hydrogen bond donors and acceptors than any other NB and has been shown to form homochiral domains on various surfaces. It has four hydrogen atoms that potentially can act as H bond donors (H_1_, H_2a_, H_2b_, and H_9_) to the ring oxygen atom of ribose. The analysis of models clearly shows that H_2a_ and H_2b_ are located too far away, i.e., if the ribose ring oxygen atom forms a hydrogen bond with any of the H_2_ guanine atoms, then some of the ribose OH groups cannot become hydrogen bond donors to guanine nitrogen or oxygen atoms. Thus, only H_1_ and H_9_ can be considered as candidates for hydrogen bond formation with the ribose ring oxygen atom.

Besides the oxygen atom of guanine, all its nitrogen atoms can act as hydrogen bond acceptors (with a possible exception of N_9_). Also, all the nitrogen-bound hydrogen atoms can act as H-bond donors. We consider a plausible formation of homochiral domains with the help of hydrogen bonds between guanine molecules. The resulting planar and homochiral surface has still an excess of H bond centers to interact with ribose (see Figure 4). However, it must be emphasized that some of the atoms involved in the hydrogen bonds between guanine molecules may also participate in the hydrogen bonding between ribose and guanine molecules. Arguably, once the homochiral domains are formed, some of the hydrogen bonds between guanine units may be no longer necessary. Note that one ribose molecule interacts with one molecule of guanine. Consequently, a guanine quartet can adsorb four ribose molecules of the same chirality.

Consider the plausible interactions of α-ribopyranose with surface guanine. As was discussed earlier, one of the hydrogen bonds must be formed by the ribose ring oxygen atom. This establishes one interaction point between the ribose and guanine molecules. Let us discuss other possible hydrogen bonds. Assume that guanine is located on the surface, as shown on Figure 6A. Then **α-D-ribopyranose** may form hydrogen bonds involving the ring oxygen atom and the following guanine nitrogen atoms:

If ribose ring O is H-bonded to guanine H_1_, **OH1-N_3_, OH2-N_9_(O2-H_9_)**, **OH4-N_7_.**

If ribose ring O is H-bonded to guanine H_9_, **OH1-N_7_, OH3-N_1_, OH4-N_3_.**

Let us consider H-bonds between **α-L-ribopyranose** and guanine:

If ribose ring O is H-bonded to guanine H_1_, **OH1-O_6_, OH2-N_7_, OH3-N_9_** (**O3-H_9_**), **OH4-N_3_.**

If ribose ring O is H-bonded to guanine H_9_, **OH1-N_3_**, **OH2-N_1_**, **OH3-O_6_**, **OH4-N_7_.**

Of course, if the guanine molecule was rotated by 180° around the axis formed, for example, by a C_4_-C_5_ double bond (which would result in the opposite chirality on the surface) the result would be the opposite (Figure 6B). In either case, one of the ribose enantiomers should form four hydrogen bonds with the 2D chiral surface coated with guanine, while the other enantiomer should form five hydrogen bonds. The analysis seems to indicate that the adsorption energy difference between ribose opposite enantiomers is of the order of one hydrogen bond. The average energy of a hydrogen bond for O-H---:O is 21 kJ/mol and for O-H---:N, it is 29 kJ/mol. However, note that we had to make certain assumptions here such as the favored conformations of ribose on the guanine-coated surface in a given solvent and the favored tautomers of guanine. Thus, caution about the conclusions is warranted here.

Another intriguing option is a chiral surface created by the surface adsorption of a guanine–cytosine base pair. In canonical Watson–Crick base pairing both in RNA and DNA, guanine (G) forms a base pair with cytosine (C) using three hydrogen bonds. While interactions between GC bases have been studied [83,84], there are no data on the interactions of GC bases with surfaces.

H-bonds are not the only possible interactions between ribose and the surface. For example, ribose forms exceptionally strong complexes with Ca^2+^ ions in solutions. It seems conceivable that the interactions with the surface may be of the sugar–Ca^2+^ complex type. We have previously discussed this possibility [22].

At this time, it is a matter of speculation how much time may have passed after the formation of large homochiral surfaces until racemic ribose solution came into contact with the discussed surface(s) leading to its enantioseparation.

In order to get more insight into the proposed mechanism of enantioseparation, we initiated a program to calculate the energies of interactions of L- and D-ribose with chiral surfaces, as well as of the formation of larger nucleobase-coated surfaces.

## 6. Conclusions

Towards the end of the previous century, Albert Eschenmoser [8] asked important questions concerning the structure of nucleic acids, namely why pentose, if pentose, why ribose and if ribose, and why ribofuranose. We have previously shown [22] that α-ribopyranose is the easiest monosaccharide to be separated in the absolute enantioselective separation (AES) process. Thus, arguably, nature selected ribose because it was the only monosaccharide that could be enantioseparated. We do not have an answer to the question concerning ribofuranose. The present paper offers a possible surface the AES of D,L-ribose could have taken place on.

Nucleobases are prochiral. Consequently, when a nucleobase is adsorbed on a surface, the surface becomes 2D chiral. Through hydrogen bond interactions, large homochiral domains may be formed. Another type of chiral surface may be created as a result of the adsorption of base pairs such as guanine–cytosine. We argue that a nucleobase adsorbed at the surface could have served as a chiral surface at which an AES process of a mixture of racemic monosaccharides took place. One of the ribose enantiomers was separated from its enantiomer and other racemic monosaccharides.

## Data Availability

The original contributions presented in this study are included in the article. Further inquiries can be directed to the corresponding authors.
